# The Effect of Anthropometric Measurements on the Incidence of Atrial Fibrillation in Patients With Acute Myocardial Infarction

**DOI:** 10.7759/cureus.25356

**Published:** 2022-05-26

**Authors:** Tulay Oskay, Yasin Turker, Alten Oskay, Hatem Ari, Mehmet Ozaydin

**Affiliations:** 1 Department of Cardiology, Bucak State Hospital, Burdur, TUR; 2 Department of Cardiology, Meddem Hospital, Isparta, TUR; 3 Department of Emergency Medicine, Pamukkale University, Denizli, TUR; 4 Department of Cardiology, Farabi Hospital, Konya, TUR; 5 Department of Cardiology, Akdeniz Sifa Hospital, Antalya, TUR

**Keywords:** waist circumference, obesity, myocardial infarction, body mass index, atrial fibrillation, anthropometric measurements

## Abstract

Background and objective

Atrial fibrillation (AFib) is the most common supraventricular arrhythmia occurring after myocardial infarction (MI). Height, body weight, waist and hip circumference, and body mass index (BMI) are considered potential risk factors for the development of AFib. The aim of this study was to investigate the effect of BMI and waist circumference on the incidence of AFib in patients with acute MI.

Methods

This prospective, cross-sectional, observational study was conducted in the coronary intensive care unit (CICU) of a tertiary care university hospital between July 2014 and February 2016. Patients diagnosed with ST-elevation myocardial infarction (STEMI) or non-ST-elevation myocardial infarction (NSTEMI) were included. Demographic, clinical, echocardiographic, and laboratory data, past medical history, and anthropometric measurements were recorded. Continuous electrocardiography (ECG) monitoring was performed for following up on the occurrence of AFib. Then, AFib predictors were identified using multiple regression analysis.

Results

AFib developed in 31 (9.3%) patients in the cohort. No significant difference was observed between patients with or without AFib in terms of BMI and waist circumference values (p=0.686 vs. p=0.728, respectively). Factors associated with AFib development as per the multivariate analyses included age (OR: 1.051, 95% CI: 1.013-1.09; p=0.008), pulse rate (OR: 1.043, 95% CI: 1.018-1.069; p=0.001), peak troponin T value (OR: 1.356, 95% CI: 1.135-1.619; p=0.001), and length of CICU stay (OR: 2.247, 95% CI: 1.163-4.340; p=0.016).

Conclusion

BMI and waist circumference measurements were similar in patients with and without AFib during acute MI. Age, pulse rate, peak troponin T, and duration of CICU stay were identified as independent predictors of AFib development.

## Introduction

Affecting 1-2% of the general population, atrial fibrillation (AFib) is the most common cardiac rhythm disorder, the prevalence of which increases with age and reaches up to 5-15% by the age of 80 years [1]. This disorder develops at a rate of 2-21% after an acute coronary syndrome and is the most common supraventricular arrhythmia occurring after myocardial infarction (MI) [1]. Besides, the incidence of new-onset AFib during acute MI in the hospitalization period was found to be around 6-8% [2]. Previous studies have associated post-MI AFib with ischemia, decreased atrial blood flow, elevated left ventricular end-diastolic pressure, and left atrial pressure, as well as impaired diastolic and autonomic functions. Post-MI AFib is also considered to be an independent indicator of mortality [2].

Height, body weight, waist and hip circumference, and body mass index (BMI) are considered potential risk factors for the development of AFib [3]. In this regard, obesity is believed to act as an independent risk factor for both coronary artery disease and AFib development [3,4]. AFib is also associated with left atrial dilatation, left ventricular dysfunction, and hypertension [5]. Increased body size and BMI are linked to an increase in left atrial diameter and left atrial volume [6]. Obesity may also lead to left atrial dilatation, left ventricular dysfunction, and hypertension [5]. Moreover, obesity is associated with obstructive sleep apnea syndrome, diabetes mellitus, and coronary artery disease, all of which have been shown to be significant risk factors for the development of AFib [4]. However, the correlation between obesity and AFib cannot be explained based on these conditions alone, and the effect of these risk factors on AFib development during MI still remains unknown. In this study, we aimed to explore the effect of BMI and waist circumference on the development of AFib in patients with acute MI.

The abstract of this article was previously presented as a poster at the 33rd Turkish Cardiology Congress in 2017.

## Materials and methods

Study design and setting

This prospective, cross-sectional, observational study was carried out in the coronary intensive care unit (CICU) of the Süleyman Demirel University Hospital between July 2014 and February 2016. The study was performed in line with the principles of the Declaration of Helsinki. Ethical approval was granted by the Medical Research Ethics Committee of Süleyman Demirel University (72867572-050/2033). Written informed consent was obtained from all participants.

Study population

The study included 360 adult patients diagnosed with ST-elevation myocardial infarction (STEMI) or non-ST-elevation myocardial infarction (NSTEMI). The exclusion criteria were as follows: unstable angina pectoris, moderate-severe heart valve disease, left atrial diameter >45 mm, hyperthyroidism, advanced chronic obstructive pulmonary disease, sepsis, diagnosis of malignancy, being on pro-arrhythmic drug therapy, and previous AFib history. After excluding 25 patients based on these criteria, 335 patients were included for the final analysis.

We recorded the patients’ demographic, clinical, and laboratory data, their previous medication history, and the medications that we started during the CICU stay. The diagnosis of MI, hypertension, diabetes mellitus, and hyperlipidemia were made based on the current guidelines [7-10]. The treatment of all the patients was also performed as per the currently published guidelines.

At the time of CICU admission, the initial electrocardiography (ECG) was performed for all the patients. During their stay in the CICU, continuous ECG monitoring was performed for following up on the occurrence of AFib, an irregular narrow QRS complex arrhythmia with the absence of P waves. The endpoint in the study was the development of AFib episodes lasting more than 30 seconds as detected on continuous ECG monitoring or ECG during the CICU stay. Transthoracic echocardiography was performed on all the patients. Left ventricular ejection fraction (LVEF) was calculated using Simpson's method, and left atrial diameter was measured from the parasternal long-axis and the apical four-chamber views.

Anthropometric measurements

All the participating patients were weighed by using scales with bare feet and light clothes on them, and their height was measured with a tape measure. Their BMI was calculated by dividing weight by the square of height (kg/m²). Patients with a BMI of 25 and above were recorded as overweight or obese, and those with BMI below 25 as non-overweight or non-obese [11].

The waist circumference of the patients was measured in the upright standing position, after a normal expiration, from the middle of the line connecting the spina iliaca anterior superior with the lowest rib, and from the line where the waist was widest. Waist circumference >102 cm in men and >88 cm in women were considered an indicator of abdominal obesity [12].

Statistical analysis

The SPSS Statistics software version 25 (IBM Corp., Armonk, NY) was used to perform all analyses. Continuous variables were expressed as means and standard deviations (SD) and median (range); categorical variables were expressed as counts and percentages. Shapiro-Wilk and Kolmogorov Smirnov tests were used for testing normality. For independent group comparisons, we used the independent-samples t-test when parametric test assumptions were provided, and the Mann-Whitney U test was used when parametric test assumptions were not provided. Differences between categorical variables were assessed using the chi-square test. To determine the factors that were affecting AFib development results, we used univariate and multiple logistic regression models. A p-value ≤0.05 was considered statistically significant.

## Results

The mean age of 335 patients was 63 ±13 years (range: 28-91 years), and 75 (22.6%) of them were women. While the mean BMI was 27 ±4 kg/m² (range: 16-47 kg/m²) for all the patients, their mean waist circumference measured 100 ±12 cm (range: 65-138 cm). As for the risk factors of cardiovascular disease, there were 168 (50.1%) patients with a smoking habit, 80 (23.9%) with a history of diabetes mellitus, 166 (49.6%) with hypertension, and 67 (20%) with a background of hyperlipidemia.

Of note, 219 (65.4%) patients were diagnosed with STEMI and 116 (34.6%) with NSTEMI, whereas 105 (31.3%) patients were followed up with the diagnosis of anterior MI and 114 (34%) with non-anterior MI. The mean follow-up time in the CICU was 2.1 ±0.6 days. The mean left atrial diameter was 36 ±4 mm, while the mean LVEF was 45 ±9%. The demographic, clinical, and laboratory characteristics of the patients are presented in Table 1.

**Table 1:** Demographic, clinical, and laboratory data of patients classified as per a BMI of 25 ACE: angiotensin-converting enzyme; ARB: aldosterone receptor blocker; BMI: body mass index; CICU: coronary intensive care unit; CKMB: Creatinine kinase-myocardial band; MI: myocardial infarction; NSTEMI: non-ST-elevation myocardial infarction; SD: standard deviation; STEMI: ST-elevation myocardial infarction

Variables		BMI ≥25, (n=219)	BMI <25, (n=116)	P-value
Age, years, mean ± SD		63 ± 12	64 ± 15	0.655
Female gender, n (%)		58 (26.5)	17 (14.7)	0.013
Waist circumference, cm, median (range)		105 (74-138)	89.5 (65-108)	<0.001
Abdominal obesity, n (%)		146 (66.7)	11 (9.5)	<0.001
Smoking, n (%)		96 (43.8)	72 (62.1)	0.001
Diabetes mellitus, n (%)		65 (29.7)	15 (12.9)	0.001
Hypertension, n (%)		121 (55.3)	45 (38.8)	0.004
Hyperlipidemia, n (%)		57 (26.0)	10 (8.6)	<0.001
Pulse rate, beats/minute, median (range)		79 (38-160)	75.5 (48-113)	0.019
Systolic blood pressure, mmHg, median (range)		135 (80-213)	127.5 (75-190)	<0.001
Diastolic blood pressure, mmHg, median (range)		80 (40-140)	77 (32-110)	0.085
Left ventricular ejection fraction, %, median (range)		45 (20-65)	45 (20-60)	0.917
Left atrial diameter, mm, median (range)		37 (26-55)	35 (25-45)	<0.001
Left atrial width, mm, median (range)		37 (28-58)	35 (26-46)	<0.001
Left atrial length, mm, median (range)		41 (32-62)	38 (32-60)	<0.001
Duration of chest pain, hours, median (range)		5 (1-72)	5 (1-72)	0.134
MI type	STEMI, n (%)	133 (60.7)	86 (74.1)	0.014
	NSTEMI, n (%)	86 (39.3)	30 (25.9)	
STEMI	Anterior, n (%)	67 (50.4)	38 (44.2)	0.371
	Non-anterior, n (%)	66 (4.6)	48 (55.8)	
Peak CKMB, U/l, median (range)		74 (11-1,064)	119.5 (20-772)	0.007
Peak troponin T, ng/ml, median (range)		1.8 (0.05-10)	2.39 (0.05-10)	0.041
Pre-hospitalization treatment	Beta-blockers, n (%)	46 (21.0)	16 (13.8)	0.106
	Calcium-channel blockers, n (%)	28 (12.8)	8 (6.9)	0.098
	ACE inhibitors, n (%)	32 (14.6)	12 (10.3)	0.271
	ARB, n (%)	40 (18.3)	12 (10.3)	0.057
	Potassium-sparing diuretics, n (%)	5 (2.3)	2 (1.7)	1.000
	Thiazide diuretics, n (%)	39 (17.8)	15 (12.9)	0.248
	Statins, n (%)	29 (13.2)	8 (6.9)	0.078
	Acetyl salicylic acid, n (%)	42 (19.2)	19 (16.4)	0.528
	Clopidogrel, n (%)	13 (5.9)	3 (2.6)	0.171
Length of stay in CICU, days, median (range)		2 (1-5)	2 (1-6)	0.411

Overweight or obese patients were predominantly female and had higher rates of abdominal obesity, diabetes mellitus, hypertension, and hyperlipidemia (all p<0.05). Waist circumference, pulse rate, and systolic blood pressures of the overweight or obese patients were higher than those of their non-overweight or non-obese counterparts (all p<0.05). The transthoracic echocardiography findings revealed that the mean left atrial diameters were significantly higher in the overweight or obese patients than in their non-overweight or non-obese counterparts (all p=0.001) (Table 1). The treatment of the overweight or obese patients and their counterparts before hospitalization was similar (all p>0.05) (Table 1).

Among overweight or obese patients, 133 (60.7%) were followed up with the diagnosis of STEMI and 86 (39.3%) with NSTEMI. STEMI was more common in overweight or obese patients; on the other hand, NSTEMI was found to be more frequent in the non-overweight or non-obese group. The localization of STEMI (anterior vs. non-anterior) was similar between both groups (both p>0.05). All the other demographic, clinical, and laboratory features, treatment methods of the patients, and their duration of hospitalization in CICU were similar between the two groups (all p>0.05).

Out of 31 (9.3%) patients with AFib, 22 (10.0%) were overweight or obese, while nine (7.8%) were non-overweight or non-obese. The incidence of AFib in both groups was similar (p>0.05). Also, there was no difference between the two groups with respect to the duration from the onset of symptoms to AFib development, the time period during which patients suffered AFib, and methods employed to achieve sinus rhythm (all p>0.05) (Table 2).

**Table 2:** Characteristics of patients with atrial fibrillation AFib: atrial fibrillation; AFib development time: time from the start of chest pain until AFib development; BMI: body mass index

Variable		BMI ≥25 (n=219)	BMI <25 (n=116)	P-value
AFib, n (%)		22 (10.0)	9 (7.8)	0.492
AFib development time, hours, median (range)		7 (3-87)	21 (6-41)	0.219
AFib duration, hours, median (range)		5.5 (1-24)	10 (1-45)	0.203
Method of return to sinus rhythm	Spontaneous, n (%)	13 (59.1)	2 (22.2)	0.089
	Amiodarone, n (%)	8 (36.4)	7 (77.8)	
	Electrical, n (%)	1 (4.5)	0 (0.0)	

The demographic, clinical, and laboratory information of patients with and without AFib are provided in Table 3.

**Table 3:** Demographic, clinical, and laboratory information of patients with and without atrial fibrillation ACE: angiotensin-converting enzyme; AFib: atrial fibrillation; ARB: aldosterone receptor blocker; BMI: body mass index; CICU: coronary intensive care unit; CKMB: creatinine kinase-myocardial band; MI: myocardial infarction; NSTEMI: non-ST-elevation myocardial infarction; SD: standard deviation; STEMI: ST-elevation myocardial infarction

Variable			With AFib, (n=31)	Without AFib, (n=304)	P-value
Age, years, mean ± SD			71.5 ± 10.3	62.4 ± 13.7	0.001
Female gender, n (%)			9 (29.0)	66 (21.7)	0.352
BMI ≥25			22 (71.0)	197 (64.8)	0.492
Abdominal obesity, n (%)			15 (48.4)	142 (46.7)	0.859
Waist circumference, cm, mean ± SD			99.4 ± 13.9	100.18 ± 12.5	0.728
BMI, kg/m², median (range)			26.48 (16.78-40.44)	26.78 (16.07-47.05)	0.686
Smoking, n (%)			13 (41.9)	155 (51.0)	0.337
Diabetes mellitus, n (%)			5 (16.1)	75 (24.7)	0.288
Hypertension, n (%)			19 (61.3)	147 (48.4)	0.170
Hyperlipidemia, n (%)			4 (12.9)	63 (20.7)	0.300
Pulse rate, beats/minute, median (range)			84 (48-160)	77 (38-141)	0.046
Systolic blood pressure, mmHg, median (range)			129 (75-210)	132 (75-213)	0.842
Diastolic blood pressure, mmHg, median (range)			77 (43-140)	80 (32-140)	0.924
Left ventricular ejection fraction, %, median (range)			40 (20-55)	45 (20-65)	0.009
Left atrial diameter, mm, median (range)			36 (27-42)	36 (25-55)	0.771
Left atrial width, mm, median (range)			36 (27-42)	37 (26-58)	0.696
Left atrial length, mm, median (range)			42 (34-55)	40 (32-62)	0.146
Pain duration, hours, median (range)			5 (1-72)	5 (1-72)	0.987
MI type	STEMI, n (%)		25 (80.6)	194 (63.8)	0.061
	NSTEMI, n (%)		6 (19.4)	110 (36.2)	
STEMI	Anterior, n (%)		12 (48.0)	93 (47.9)	0.995
	Non-anterior, n (%)		13 (52.0)	101 (52.1)	
Peak CKMB, U/l, median (range)			287 (24-886)	80.5 (11-1064)	0.000
Peak troponin T, ng/ml, median (range)			6.86 (0.05-10)	1.7 (0.05-10)	0.000
Pre-hospitalization treatment		Beta-blockers, n (%)	6 (19.4)	56 (18.4)	0.899
		Calcium-channel blockers, n (%)	4 (12.9)	32 (10.5)	0.759
		ACE inhibitors, n (%)	3 (9.7)	41 (13.5)	0.781
		ARB, n (%)	8 (25.8)	44 (14.5)	0.116
		Potassium-sparing diuretics, n (%)	0 (0.0)	7 (2.3)	1.000
		Thiazide diuretics, n (%)	6 (19.4)	48 (15.8)	0.609
		Statins, n (%)	3 (9.7)	34 (11.2)	1.000
		Acetyl salicylic acid, n (%)	6 (19.4)	55 (18.1)	0.862
		Clopidogrel, n (%)	2 (6.5)	14 (4.6)	0.651
Length of stay in CICU, days, median (range)			2 (2-6)	2 (1-5)	0.000

The mean age, pulse rate, peak troponin T, peak CKMB, and length of stay in CICU of patients were higher and the LVEF was lower in patients who developed AFib compared to those without AFib (all p<0.05). The treatment of the patients in whom AFib was detected and their counterparts before hospitalization was similar (p>0.05, Table 3). Other demographic, clinical, laboratory, and electrolyte values were similar between both groups (all p>0.05).

In the univariate logistic regression examination, it was observed that the variables of age, pulse rate, peak troponin T, LVEF, peak CKMB, and duration of CICU stay increased the risk of AFib in a statistically significant way. As per the multiple logistic regression analysis established with these variables, it was determined that the main effective risk factors were age, pulse rate, peak troponin T values, and length of stay in CICU. These four variables were found to significantly increase the risk of AFib development (Table 4).

**Table 4:** Independent predictors of atrial fibrillation development CICU: coronary intensive care unit; CKMB: creatinine kinase-myocardial band

		P-value	OR	95% CI for OR	
				Lower	Upper
Univariate analysis	Age	0.001	1.060	1.025	1.095
	Pulse rate	0.01	1.029	1.007	1.051
	Peak troponin T	<0.001	1.378	1.232	1.542
	Left ventricular ejection fraction	0.004	0.948	0.913	0.983
	Peak CKMB	<0.001	1.004	1.002	1.006
	Length of stay in CICU	<0.001	3.749	2.128	6.605
Multivariate analysis	Age	0.008	1.051	1.013	1.09
	Pulse rate	0.001	1.043	1.018	1.069
	Peak troponin T	0.001	1.356	1.135	1.619
	Left ventricular ejection fraction	0.57	1.014	0.967	1.062
	Peak CKMB	0.885	1.000	0.997	1.003
	Length of stay in CICU	0.016	2.247	1.163	4.34

## Discussion

Obesity is responsible for an elevation in heart rate, stroke volume, and blood pressure parameters through various hemodynamic mechanisms [13,14]. Increased blood volume and left ventricular diastolic pressure lead to left atrial dilatation in line with an elevation in left ventricular volume, which plays a key role in the AFib pathogenesis [15].

Wang et al.’s study reported a 50% increase in AFib risk in obese patients compared to those with normal BMI [16]. Given the cardiovascular risk factors, the risk of AFib was observed to increase by 4% for each unit increase in BMI [16]. In a study published in 2016 on BMI and newly developed AFib risk in middle-aged adults, 18,290 cases were followed for an average of six years, and it was found that being obese and overweight was associated with an increased risk of AFib [17]. In a randomized controlled study with 248 patients, the frequency of AFib episodes and AFib recurrences decreased proportionately with weight loss [18]. Similarly, Pathak et al. reported six times more arrhythmia-free survival through regular weight loss in their study cohort during a five-year follow-up [19].

A rise in BMI is also associated with left atrial dilatation, which shows a significant correlation with the increased risk of AFib [20]. In obese patients, left atrial dilatation may develop due to elevated central blood volume, left ventricular diastolic pressure, elevated left ventricular volume, and heart failure [15]. The increase in left atrial pressure due to left ventricular diastolic dysfunction might also increase atrial ectopia, triggering the development of AFib.

Exploring the effect of body weight and composition on cardiac structure and functions, the ARIC study in 2016 reported that the presence of obesity was not correlated with the LVEF, an indicator of left ventricular systolic functions, which is similar to our study. In the same study, a significant increase in left atrial volume was observed in parallel to increasing obesity levels in both genders [21]. In another study consisting of 1,000 STEMI cases, the LVEF was lower in the patients with AFib compared to those without AFib, and the left atrial diameters were significantly higher [22]. Also, in a study by Asanin et al., LVEF was significantly lower in patients with AFib after MI than in those without AFib [23]. Likewise, LVEF was lower in the group with AFib, but the presence of left atrial dilatation made the study different from ours.

We evaluated the effect of BMI and waist circumference among obesity parameters on AFib during MI. We divided our study patients into two groups: non-overweight and overweight or obese in accordance with the current WHO guidelines [11]. In a study of 1,193 patients with type 2 diabetes mellitus and acute coronary syndrome, the mean age was reported to be 64.1 years, which is similar to our study. Patients with a BMI of 24 and above were defined as overweight and obese. While hypertension and dyslipidemia history turned out to be significantly higher in overweight and obese patients in that study, as in our study, there was no significant difference in terms of smoking between the groups [24].

We identified age, pulse rate during hospitalization in CICU, peak troponin T, and length of stay in CICU as independent predictors of AFib development. This finding is supported by Aksoy et al.’s study, published in 2019, in which the CHA2DS2-VASc score was found to be a predictor of AFib development in STEMI patients [25]. On the contrary, in our study, BMI and waist circumference were not associated with the development of AFib. In a study by Guenancia et al., obesity was independently associated with newly developing AFib only in male patients, and age was independently associated with AFib development [26]. Their study patients were followed up during their hospital stay [24]. It is plausible that the more prolonged the follow-up process, the more likely it is to detect arrhythmic cases. On the other hand, our follow-up period was confined to the duration of our patients' stay in the ICU. If the study population had been larger and the patients had been followed up by continuous monitoring in the cardiology service, we might have found an association between AFib and obesity.

The factors associated with AFib development in the course of MI have been explored in a large body of research before. For example, advanced age, male gender, increased heart rate (>100 beats/minute), and the Killip class IV turned out to be independent predictors of AFib development in the OACIS study, in which 7.7% of the patients followed up with STEMI were reported to develop AFib during their hospital follow-up [27]. Similar to that study, we have found that a high pulse rate is an independent predictor of the development of AFib.

In the TRACE study, 21% of 6,676 acute MI patients with LVEF ≤36% developed AFib during their follow-up process, increasing hospitalization time by 1.5 times and causing a significant increase in mortality during the in-hospital period and five-year follow-up. In that study, age, female gender, previous heart failure history, smoking, absence of thrombolytic treatment, hypertension, and diabetes mellitus were found to be independent predictors of AFib development [28]. In our study, patients with AFib tended to stay longer in CICU than those without AFib. Although post-acute MI length of stay in hospital has been reported to be shortened in recent years, AFib development in patients with acute MI may extend these periods [29].

In the APEX-MI study, which included STEMI patients undergoing primary percutaneous intervention, CKMB, troponin, and B-type natriuretic peptide levels were significantly higher in patients with AFib compared to those without AFib [30]. Peak troponin T value was also found to be an independent predictor of AFib development in our study.

In a previous study by our group, involving 1,000 patients with acute coronary syndromes, AFib developed in 8.8% of the patients during the hospitalization period. As per the findings of that study, the independent predictors of AFib development were age, left atrial diameter, hypertension, prior statin and renin-angiotensin system blocker use, and prior AFib history [31]. With our current study, we took these data one step further.

We are aware that a few limitations may have influenced the results obtained in this study. Firstly, ours was not a randomized study. Second, only BMI and waist circumference were taken into account as obesity parameters. Other anthropometric measurements, such as hip circumference or waist/hip ratio, were not included for evaluation within the framework of this study. Third, patients with unstable angina pectoris were excluded because the diagnosis of unstable angina pectoris depends on subjective criteria such as chest pain and the duration of angina pectoris. Finally, the major limitation might be the implementation of rhythm follow-up only in the CICU, but not in the cardiology service. However, the plausible reason for not considering service follow-ups is that continuous monitoring is not a routine practice for ward hospitalizations.

## Conclusions

Despite the fact that height, body weight, waist and hip circumference, and BMI are associated with AFib, and that AFib is a common rhythm disorder after acute MI, the effect of these risk factors on AFib development during MI still remained unknown. The present study showed that BMI and waist circumference measurements are similar in patients with and without AFib during acute STEMI or NSTEMI. Additionally, age, pulse rate per minute, peak troponin T, and length of stay in CICU are independent predictors of AFib development.
